# Theoretical comparison of two setups for capillary pressure measurement by centrifuge

**DOI:** 10.1016/j.heliyon.2022.e10656

**Published:** 2022-09-21

**Authors:** Jassem Abbasi, Pål Østebø Andersen

**Affiliations:** a Department of Energy Resources, University of Stavanger, 4036, Norway; b The National IOR Centre of Norway, University of Stavanger, 4036, Norway

**Keywords:** Special core analysis (SCAL), Centrifuge capillary pressure measurement, One-End-Open (OEO), Counter-current flow

## Abstract

There are several approaches for the calculation of capillary pressure curves in porous media including the centrifuge method. In this work, a new installation of centrifuge test is introduced and compared with the traditional setup. In the first setup, which is a standard approach in labs, the core face closest to the rotational axis is open to the non-wetting phase, while the farthest face is open to the wetting phase where strictly co-current flow is generated in rotations; labeled Two-Ends-Open (TEO). In the second setup, which is proposed as a new approach, only the outer radius surface is open and is exposed to the light non-wetting phase; labeled One-End-Open (OEO). This setup strictly induces counter-current flow. The two systems and their corresponding boundary conditions are formulated mathematically and solved by a fully implicit numerical solver. The TEO setup is validated by comparison with commercial software. Experimental data from the literature are used to parameterize the models. It is mathematically, and with examples, demonstrated that the same equilibrium is obtained in both systems with the same rotational speed, and changing the installation does not influence the measured capillary pressure. This equilibrium state is only dependent on the rotational speed, rock capillary pressure properties, and fluid densities, not the installation geometry, relative permeabilities, or fluid viscosities. However, the dynamic transition trend and saturation profiles were found to be dependent on the applied installation. It was observed that the OEO setup takes almost identical equilibration time as the TEO setup for mixed-wet states, although it needed much longer time in water-wet states. The presence of threshold capillary pressure significantly increased the time scale of the OEO setup. Also, it was found that in contradiction to the TEO setup, the dynamic saturation profile in OEO was rarely influenced by viscosity ratio. To conclude, the performed history matching analysis demonstrated that the OEO setup can be applied for the calculation of counter-current relative permeability from the production data with reasonable accuracy.

## Highlights

• It is demonstrated that the same equilibrium was obtained in both OEO and TEO systems.
• Only for the TEO case, the dynamic saturation profile was influenced by the viscosity ratio of fluids.
• This OEO setup should help to measure counter-current relative permeability by an inverse calculation method.
• Our analysis of the OEO setup showed that obtaining a unique relative permeability is guaranteed under different conditions.

## 1 Introduction

The flow in porous media has applications in several scopes of nature and industry like environmental engineering, carbon storage, microfluidics, and hydrocarbon reservoirs (Blunt, 2017). The capillary pressure function is a key parameter in the modeling of multiphase flow phenomena in porous media. Especially, it controls fluid distributions established over geological time due to the balance between gravity and capillary forces, but also plays a key role in the recovery of hydrocarbons in naturally fractured reservoirs where spontaneous imbibition is perhaps the most important recovery mechanism (Andersen et al., 2014; Mason and Morrow, 2013). Capillary pressure can also affect the measurement and interpretation of relative permeability from core flooding experiments (Andersen, 2021; Moghaddam and Jamiolahmady, 2019; Rapoport and Leas, 1953; Richardson et al., 1952). The drainage capillary pressure characterizes the pore-size distribution and the ability of the porous medium to resist or engage the uptake of one fluid while expelling another. The relative movement of fluids with respect to each other creates two different regimes of flow called co-current flow (both fluids flow in the same direction) and counter-current flow (fluids flow in opposing directions). This flow regime is reported to be effective in the multiphase flow properties of rock such as relative permeability (Javaheri and Jessen, 2011). In the counter-current flow regime, the relative permeabilities of fluids in the same porous media are often lower than co-current flow (Haugen et al., 2015), which can be related to the reduction in the driving forces per unit volume of the rock (Bentsen and Trivedi, 2013). Andersen et al. (2019) related this reduction of relative permeability to the viscous coupling effects and the strength of fluid-fluid interactions.

There are several experimental methods for the calculation of capillary pressure curves in the laboratory including the centrifuge method, porous plate, and mercury injection or their combination with technologies like nuclear magnetic resonance techniques (Karimi et al., 2017; Ruth and Chen, 1995). This work aims at providing a new setup of the centrifuge system that strictly induces a counter-current flow regime. As a routine approach in labs, a two-end open (TEO) geometry is used. This approach, during a drainage experiment, typically consists of rotating a core plug at a fixed rotational speed and seeing how much wetting phase is expelled from one side, while a corresponding amount of nonwetting phase enters from the other side. By increasing the rotational speed, a more volume of the wetting phase is expelled. The amount is determined by how strongly the capillary forces are able to hold the wetting phase and as capillary pressure increases with lower saturation it takes a higher rotation speed to reduce the saturation further. The distribution of phases at equilibrium between the centrifugal forces and capillary forces is non-uniform and was described by Hassler and Brunner (1945). Methods suggested for the interpretation of centrifuge data can be found in the work of Nazari Moghaddam (2015). The most popular methodology for interpretation of the centrifuge data is from Forbes (1990) which helps in easy calculation of local saturations from volumetric production data. An analytical evaluation of the centrifuge drainage test in the TEO setup was introduced by Andersen et al. (2020) to derive a time scale and recovery function between two rotation speeds. Ferno et al. (2007) showed that the time to reach equilibrium in routine experimental tests is dependent on the permeability and wettability preference of tests.

Centrifuge tests also can be used as a wettability determination method as explained by Chen et al. (2017) and in the calculation of relative permeability by history matching of the phase depletion process (Nordtvedt et al., 1993; O'Meara Jr and Lease, 1983; van Spronsen, 1982). Tanino and Christensen (2019) indicated the importance of numerical simulation for the full interpretation of core-flood and centrifugal tests. Firoozabadi (1986), however, found it challenging to determine relative permeability and capillary pressure curves uniquely from a single centrifuge test. While the centrifuge capillary pressure tests are normally occurring in co-current mode (in the TEO system), there are several instances where counter-current flow is dominant like spontaneous imbibition in water invaded matrix/fracture systems (Abbasi et al., 2017; Qiao et al., 2018).

This work will present a new design for centrifuge capillary pressure tests that helps in the measurement of capillary pressures in a different way and also the calculation of the counter-current relative permeabilities in imbibition or drainage processes. We will compare the traditional TEO setup against the proposed one-end-open (OEO) setup in the equilibrium and dynamic states. The sensitivity analysis on the impact of different parameters such as capillary pressure threshold, viscosity ratio, and wettability will also be carried out. The practical approach for the calculation of relative permeability will be investigated and the associated uncertainties will be discussed. The paper is structured as follows: The geometry descriptions, differential equations, and different boundary conditions are presented in the theory section (Section 2). After that, in Section 3, different example cases with both boundary conditions are compared. Then, discussions about the results are provided and the paper will be finished with some conclusions. Also, complementary materials including the numerical implementation and validation of the model, plus the history matching of the production profile are presented in the appendices, at the end of the paper.

## 2 Theory

**Figure 1:**
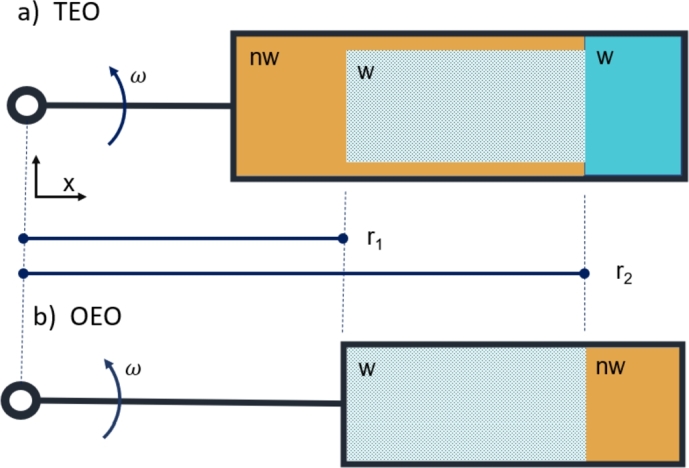
The geometry of the centrifuge setups. The core is aligned with the *x*-axis, which rotates around the center, *x* = 0, with rotational frequency *ω*. The core is mounted between *r*_1_ < *x* < *r*_2_ in a core holder and saturated with the wetting phase in both cases. a) Two ends open setup, b) One end open setup.

We consider two installations of centrifuge tests with different boundary conditions, applied to calculate drainage capillary pressure curves (primary, secondary, etc.). The following description is illustrated in Fig. 1. Common for both setups, a core plug is initially saturated with high wetting (*w*) phase saturation corresponding to zero capillary pressure. This situation corresponds to the state at primary or secondary drainage before any force is applied to displace the wetting fluid but after spontaneous drainage has allowed the sample to take up oil spontaneously. The core is located in a rotating system, on an axis termed *x* that is aligned outwards from the center of rotation, where x=0. *x* is positive outwards from the center and the core is mounted at $r_{1}<x<r_{2}$. The core is assumed sealed in the directions normal to the *x*-axis to treat the system in one dimension. The centrifugal force enforces the non-wetting phase to enter the core and displace the wetting phase. In the drainage process, the capillary pressure resists this displacement.

### 2.1 Model geometry

In the first system, termed Two-Ends-Open or TEO (see Fig. 1a), the inner boundary $x=r_{1}$ is exposed to non-wetting (*nw*) low-density phase which extends outside the core to a free w/nw surface (implemented experimentally by a water bath) at $x=r_{2}$ where $P_{c}=0$ (Forbes, 2000). The core is exposed to the wetting (dense) phase at $x=r_{2}$. Note that the *nw* phase has pressure continuity in the space outside the core from $x=r_{1}$ to $x=r_{2}$, while due to the sealed surface on the core sides the *nw* phase has pressure continuity into the core only at $x=r_{1}$. When the system begins to rotate, defined by the rotational speed *ω*, wetting phase is pushed out of the core at $r_{2}$, while non-wetting phase enters at $r_{1}$. Increasing the rotational speed further reduces the wetting phase saturation of the core. More discussions about this setup can be found in Andersen et al. (2020). In the second system, termed One-End-Open or OEO (see Fig. 1), the inner boundary at $x=r_{1}$ is closed and only the outer boundary at $x=r_{2}$ is open; in this case exposed to *nw* phase. By keeping the w/nw surface very close to $x=r_{2}$ we also assume zero capillary pressure $P_{c}=0$ at $x=r_{2}$ for this system. When this system begins rotating, the dense *w* fluid must leave through the same surface as the light *nw* fluid enters, namely at $x=r_{2}$. In both TEO and OEO setups, monitoring the volume of produced wetting phase versus time is vital. So, the calibrated transparent container is attached to the end of the core bucket ($x>r_{2}$) and the volume of fluid being expeled in this container can be recorded during the test.

### 2.2 Mass balance equations

We consider a system with immiscible and incompressible fluids, where the lighter fluid is non-wetting (e.g., oil) while the dense fluid is wetting (e.g., water). The porous medium is assumed homogeneous and incompressible. Darcy's law in a rotating system is given by (Chen et al., 2006):

(1) $$u_{i}=-\lambda_{i}[\partial_{x}p_{i}-\rho_{i}\omega^{2}x],\;\lambda_{i}=\frac{Kk_{ri}}{\mu_{i}},\;(i=w,nw),$$

where $u_{i}$ is Darcy velocity, $\lambda_{i}$ mobility, $p_{i}$ pressure, $\rho_{i}$ density, *ω* angular speed (rad/s), *K* absolute permeability, $k_{ri}$ relative permeability and $\mu_{i}$ viscosity. The index *i* refers to phase-specific properties for the wetting i=w and non-wetting i=nw phases. Transport of each phase is described by the conservation law (Chen et al., 2006):

(2) $$\phi \partial_{t}(s_{i})=-\partial_{x}(u_{i}),\;(i=w,nw),$$

where $s_{i}$ is phase saturation. The saturations are constrained by volume conservation, and the pressures are constrained by the drainage capillary pressure function:

(3) $$s_{w}+s_{nw}=1,\;p_{nw}-p_{w}=P_{c}(s_{w}).$$

The latter is assumed to be a unique function of $s_{w}$ since the saturations change monotonously with time. By adding $u_{w}$ and $u_{nw}$ and eliminating $p_{nw}$ using Eq. (3) we introduce the total Darcy velocity $u_{T}$ and total mobility $\lambda_{T}$ in Eqs. (4) and (5):

(4) $$u_{T}=u_{w}+u_{nw}=-\lambda_{nw}\partial_{x}P_{c}-\lambda_{T}\partial_{x}p_{w}+[\lambda_{nw}\rho_{nw}+\lambda_{w}\rho_{w}]\omega^{2}x,$$

(5) $$\lambda_{T}=\lambda_{w}+\lambda_{nw}.$$

By substituting Eq. (4) in Eq. (2) summed over the two phases (and considering $s_{w}+s_{nw}=1$), we obtain that the total Darcy velocity is uniform:

(6) $$\partial_{x}u_{T}=0.$$

The wetting phase *w* equation in the Eq. (2) can be expressed with variables $u_{T}$, and $s_{w}$. So, by substituting Eq. (1) in $u_{w}=u_{T}-u_{nw}$, and then substituting it in the Eq. (2) we will have:

(7) $$\phi \partial_{t}s_{w}=-\partial_{x}[\frac{\lambda_{w}}{\lambda_{T}}u_{T}+\frac{\lambda_{w}\lambda_{nw}}{\lambda_{T}}\partial_{x}P_{c}+\frac{\lambda_{w}\lambda_{nw}}{\lambda_{T}}\Delta \rho \omega^{2}x],$$

where, $\Delta \rho =\rho_{w}-\rho_{nw}>0$ is the fluid density difference. The terms on the right-hand side represent co-current flow, counter-current capillary flow, and phase separation due to rotation, respectively. In this work, the recovery factor is defined as the proportion of the water volume that is depleted from the core (in comparison to the initial volume of water in the core) during the drainage process:

(8) $$\mathrm{RF}=\frac{{\bar{s}}_{\mathrm{init}}-{\bar{s}}_{w}}{1-s_{nwr}-s_{wr}}$$

In Eq. (8), ${\bar{s}}_{\mathrm{init}}$ is the average initial wetting phase saturation, $s_{nwr}$ is residual non-wetting phase saturation and $s_{wc}$ is residual water saturation.

### 2.3 Boundary conditions

As previously the model geometry is introduced, the boundary conditions are what distinguish the TEO and OEO setups. These differences may create vast changes in the system of equations being solved. In the following sections, two boundary conditions and their mathematical representations are studied.

#### 2.3.1 TEO boundary conditions

The w/nw interface outside the core at $x=r_{2}$ defines a zero capillary pressure. For reference, both phase pressures are set to 0 there and the *w* phase pressure goes continuously into the core (Andersen et al., 2020):

(9) $$p_{nw}(r_{2}^{+},t)=0,\;p_{w}(r_{2},t)=0,\;P_{c}(r_{2}^{+},t)=0.$$

By Eq. (9) we mean that the *nw* phase does not have pressure continuity at this side and cannot be produced during drainage, hence the mobility of the *nw* phase is set to zero at this boundary:

(10) $$\lambda_{nw}(r_{2},t)=0.$$

The pressure of the non-wetting phase residing outside the core decreases hydrostatically towards the rotation axis and acts continuously into the core at the inner boundary. For a given rotation speed *ω*; $p_{nw}$ at $r_{1}$ is then given by:

(11) $$p_{nw}(r_{1}^{+})=p_{nw}(r_{1}^{-},t)=\int_{r_{2}}^{r_{1}}\rho_{nw}\omega^{2}xdx=-\frac{1}{2}\rho_{nw}\omega^{2}(r_{2}^{2}-r_{1}^{2}).$$

In Eq. (11), $p_{w}$ at $r_{1}$ follows from the capillary pressure constraint:

(12) $$p_{w}(r_{1}^{+},t)=p_{nw}(r_{1}^{+},t)-P_{c}(s_{w}(r_{1}^{+},t))=-\frac{1}{2}\rho_{nw}\omega^{2}(r_{2}^{2}-r_{1}^{2})-P_{c}(s_{w}(r_{1}^{+},t)),$$

but is not continuous with the external at $r_{1}$. Similarly, to the other boundary, the *w* phase does not have the potential to be produced from this boundary during drainage and we set:

(13) $$\lambda_{w}(r_{1},t)=0.$$

To compute solutions for the TEO system, the pressure distribution must be calculated to obtain $u_{T}$ which in turn is used to update the saturation distribution.

#### 2.3.2 OEO boundary conditions

In the OEO system the boundary at $x=r_{1}$ is closed which yields:

(14) $$u_{w}(x=r_{1},t)=u_{nw}(x=r_{1},t)=0,\;u_{nw}(x=r_{2},t)=-u_{w}(x=r_{2},t).$$

Note in particular that due to the uniformity of $u_{T}$ and the above boundary conditions we obtain:

(15) $$u_{T}=0$$

In other words, the flow is strictly counter-current. Due to the open space at $x=r_{2}$ a zero capillary pressure is assumed, which goes continuously into the core since both phases flow through this face simultaneously, though in opposite directions. Both phase pressures are also set zero there for reference.

(16) $$p_{nw}(r_{2},t)=0,\;p_{w}(r_{2},t)=0,\;P_{c}(r_{2},t)=0.$$

The mobility of the *nw* phase is based on maximum *nw* saturation since that is the surrounding phase, while the mobility of the *w* phase is based on the *w* phase saturation in the core.

(17) $$\lambda_{nw}(r_{1},t)=\lambda_{nw}(s_{wc}),\;\lambda_{w}(r_{2},t)=\lambda_{w}(s_{w}(r_{2}^{-},t)).$$

With $u_{T}$ eliminated from Eq. (7), the saturation distribution as a function of time can be solved without the pressure equation. This equation is however useful for providing the pressure distribution along with the core.

We remark that this system is similar to that of a (1D) counter-current spontaneous imbibition system where a high saturation nonwetting phase is present in the core initially and a maximum wetting phase resides outside the core. It is a standard modeling assumption to set a zero boundary capillary pressure and let the mobilities be evaluated according to where the phases travel from, especially at the boundary, to allow flowing conditions (Tavassoli et al., 2005). A numerical challenge in solving the above equations is that the zero capillary pressure boundary condition sets the mobility of the non-wetting phase to zero in strongly wetted systems that lead to no-flow conditions. This problem is overcome using the above approach.

### 2.4 Equilibrium distributions

At hydrostatic equilibrium state of fluids (for a given rotational speed), we have $\partial_{t}s_{w}=0$ and $u_{T}=0$ in Eqs (4), (6), and (7). We get a distribution of capillary pressure $P_{c}^{eq}(x)$ that must obey the following:

(18) $$0=\partial_{x}u_{w}=\partial_{x}[-\frac{\lambda_{w}\lambda_{nw}}{\lambda_{T}}\partial_{x}P_{c}^{eq}-\frac{\lambda_{w}\lambda_{nw}}{\lambda_{T}}\Delta \rho \omega^{2}x].$$

Since the wetting phase flux is 0 at equilibrium, Eq. (18) can be integrated to give:

(19) $$\partial_{x}P_{c}^{eq}=-\Delta \rho \omega^{2}x,\;\text{and}\;P_{c}^{eq}(x)=C-\frac{1}{2}\Delta \rho \omega^{2}x^{2},$$

where *C* is a constant of the second-order integration. For simplicity, it is assumed that the capillary pressure function $P_{c}(s_{w})$ is continuous to the value zero. In accordance with hydrostatic equilibrium, both phases have zero pressure at $x=r_{2}^{-}$ and hence $P_{c}^{eq}(x=r_{2})=0$. After solving Eq. (19) for *C* under this condition we obtain:

(20) $$P_{c}^{eq}(x)=\frac{1}{2}\Delta \rho \omega^{2}(r_{2}^{2}-x^{2}),$$

which is a 2^nd^ order polynomial with distance, decreasing outwards. Hence, the two systems have the same distribution of capillary pressure and saturations at the same rotational speeds, regardless of the applied boundary conditions. At $x=r_{1}$, we have:

(21) $$P_{c}^{eq}(r_{1})=\frac{1}{2}\Delta \rho \omega^{2}(r_{2}^{2}-r_{1}^{2}).$$

This is the well-known Hassler-Brunner relationship (Hassler and Brunner, 1945).

### 2.5 Solution procedure

During the forward simulation of centrifuge tests, the system of equations is solved numerically with a fully implicit (implicit pressure and implicit saturation) scheme which is outlined in detail in Appendix A. This fully implicit choice was made to ensure that secure stability with acceptable time steps is established. As shown in Appendix B, after sensitivity analysis, the used number of cells was $N_{x}=40$ in both TEO and OEO setups and time-step is changed in each step of simulation by considering the stiffness of the solver to converge in the previous step, i.e., the number of iterations. The time-step length is increased by a factor of 1.1 if the convergence had occurred in less than 3 iterations and is reduced by a factor of 0.9 if the convergence had occurred in more than 5 iterations. The initial time step is selected to be 0.001 seconds to record the beginning stages of the flow. It should be mentioned that a basic assumption of our approach is that the rotational speed is altered instantaneously at the beginning of each stage (after stabilization in the previous stage).

The fluid withdrawal rate, i.e., average saturation change rate ($\partial {\bar{s}}_{w}/\partial t$), of $1\times 10^{-10}\;(1/s)$ was used as the critical level of reaching the equilibrium condition at each rotational speed. The time that this condition is reached is defined as the equilibration time for each rotational stage. For each rotational speed, this time was enough to ensure the equilibrium of the fluids (stopping production). The code corresponding to the TEO setup had been validated against commercial software (Sendra v2018.5) to ensure that the obtained results are referable. Complementary information regarding the code is provided in Appendix B.

## 3 Results

### 3.1 Input parameters

**Figure 2:**
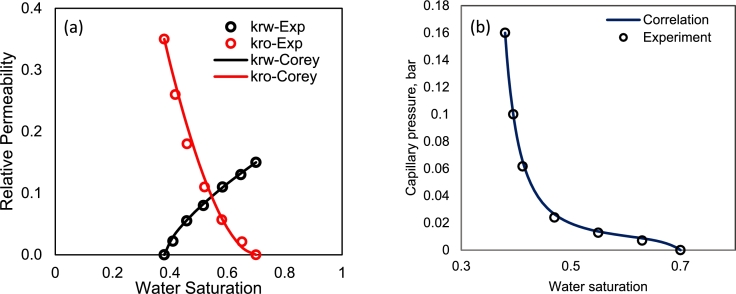
The base saturation functions used in this study. The experimental data is related to the drainage oil-water core flooding experiments from Kumar et al. (2014); points are experimental data and lines are fitted models: (a) the relative permeability data and Corey equation, (b) capillary pressure data and Andersen et al. (2017) model.

We will present simulation results considering a synthetical primary drainage test where oil displaces water from a core. Regarding the input saturation functions, we utilized Corey type relative permeabilities (Brooks and Corey, 1964) and Andersen et al. (2017) capillary pressure correlation as they are shown in Eqs. (22)–(24).

(22) $$k_{rw}=k_{rw}^{⁎}{\left(s_{w}\right)}^{n_{w}},\;k_{rnw}=k_{rnw}^{⁎}{\left(1-s_{w}\right)}^{n_{nw}},$$

(23) $$P_{c}=\frac{a_{1}}{1+k_{1}S_{w}^{n_{1}}}-\frac{a_{2}}{1+k_{2}{\left(1-S_{w}\right)}^{n_{2}}}+a_{3}$$

(24) $$S_{w}=\frac{s_{w}-s_{w,\min}}{s_{w,\max}-s_{w,\min}}$$

where $s_{w,\min}=s_{wr}$ and $s_{w,\max}=1-s_{nwr}$ denote the lowest and highest wetting phase saturation during primary drainage. In both cases we assume $s_{w,\min}=s_{wr}$. Note however that the centrifuge process only considers the forced drainage interval such that for secondary drainage the initial water saturation may be lower than $s_{w,\max}$. $n_{w}$ and $n_{nw}$ are Corey exponents and $k_{rw}^{⁎}$ and $k_{rnw}^{⁎}$ are the relative permeability endpoints. The reference parameters for rock, fluid, and saturation functions are listed in Table 1. The rock and fluid properties as well as relative permeability and capillary pressure curves are obtained from the experimental dataset reported by Kumar et al. (2014), where a drainage core flooding experiment is carried out on a Berea sandstone sample (Fig. 2). To fit the model to the capillary pressure data, we fixed the endpoints $P_{c,\max}$ and $P_{c,\min}$ at the saturation endpoints by fixing the parameters $a_{1},a_{2}$, respectively. The remaining parameters $a_{3},k_{1},k_{2},n_{1},n_{2}$ are optimized freely to minimize the error values between the model and experimental data. In the next sections, we have utilized the rotational speeds in the range of 0-50 rad/s. These values have been back-calculated from the capillary pressure curve and using Eq. (21).

**Table 1:** Rock-fluid input parameters used in the numerical simulations (Kumar et al. (2014)).

Parameters	Values	Parameters
*K*	244 mD	*r*_1_
*ϕ*	0.2	*r*_2_
*ρ*_*w*_	1.0 g/cc	$k_{rw}^{⁎}$
*ρ*_*nw*_	0.7 g/cc	$k_{rnw}^{⁎}$
*μ*_*w*_	0.7 cP	*s*_*w*,min_
*μ*_*nw*_	2.1 cP	*s*_*w*,max_
25 cm	*k*_1_	7
30 cm	*k*_2_	0.4
0.15	*n*_*w*_	0.7
0.35	*n*_*nw*_	1.8
0.35	*n*_1_	2
0.70	*n*_2_	10

**Figure 3:**
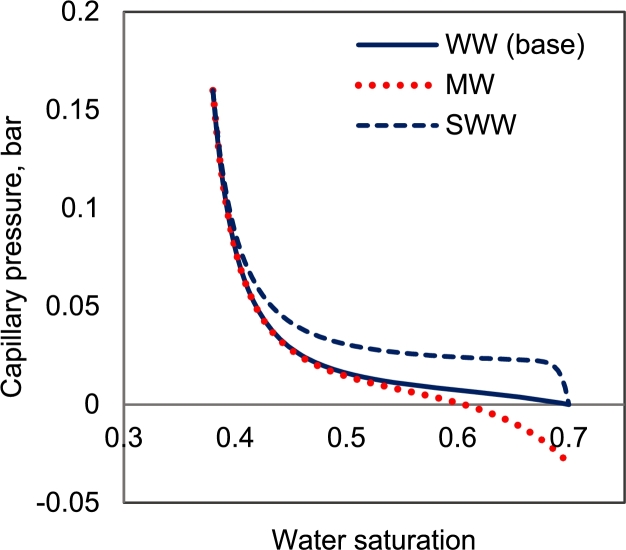
Different designed capillary pressure curves to investigate the impact of the wetting condition on the response of the centrifuge setups. The ww case is the base saturation function used in this study (Kumar et al., 2014). mw and sww cases are obtained by changing the correlation coefficients of Andersen et al. (2017) model (shown in Table 2). All other parameters including the relative permeabilities are assumed constant.

The applied relative permeabilities (Fig. 2a) are assumed to be constant in all cases (Table 1). To investigate the impact of the wetting behavior of the system on the flow regime of two setups, two mixed wet (*mw*) and strongly water-wet (*sww*) capillary pressure curves are spined off from the original curve by changing the model parameters. The capillary pressure curves are shown in Fig. 3. Also, more detailed information related to the capillary pressure model parameters is shown in Table 2.

**Table 2:** Parameters related to the capillary pressure curves used for the sensitivity analysis. Other parameters are constant between all the curves.

Parameters	Value		
Curve	Pc_ww	Pc_sww	Pc_mw
Type	Water-wet	Strongly water-wet	Mixed-wet
*P*_*c*,min_(*a*_2_)	0 bars	0.04 bars	−0.04 bars
*P*_*c*,max_(*a*_1_)	0.16 bars	0.16 bars	0.16 bars

### 3.2 Equilibrium conditions for OEO and TEO setups

**Figure 4:**
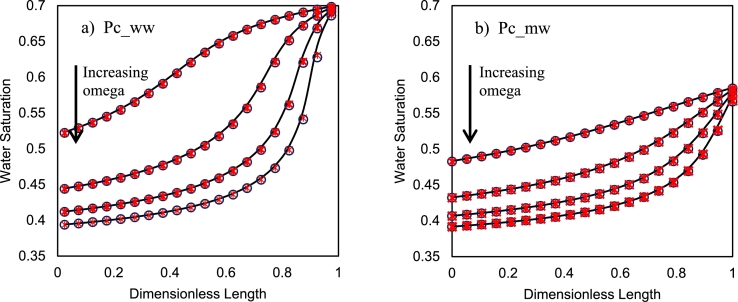
Comparison of saturation distribution at gravity-capillary equilibrium conditions for both analytical (lines) and numerical simulations (markers); black circles (**∘**) are related to OEO setup and red stars (*) are related to TEO setup. The saturation distribution curves are plotted for different rotation speeds of 20, 30, 40, and 50 rad/s, respectively.

In this part, the results of numerical simulation of centrifuge capillary pressure tests in both OEO and TEO boundary conditions, in their equilibrium state, are provided and compared. The capillary pressure curves are varied to obtain saturation distributions for different rock wettability conditions. Table 2 shows the capillary pressure correlation coefficients and Fig. 3 shows the capillary pressure curves. For sensitivity analysis purposes, the simulations were repeated with these capillary pressure curves (water-wet, *Pc_ww*, and mixed-wet, *Pc_mw*). The rotational speed was selected similarly for all cases (20, 30, 40, and 50 rad/s). The saturation distribution of phases after reaching the equilibrium is shown in Fig. 4. Observable from this figure, the phases reach the same equilibrium saturation profiles in both TEO and OEO cases at the same rotation speeds. At equilibrium conditions, the capillary and gravity forces are balanced, and the fluid movement is stopped. This result validates the hypothesis proven in section 2.5 confirming that the distribution of fluids approaches the same equilibrium states in TEO and OEO systems. The saturation of the wetting phase at $x=r_{2}$ converges to saturation corresponding to the zero capillary pressure ($P_{c}(S_{w})=0$) for each capillary pressure curve to attain a balance between gravitational (rotational) and capillary forces at the outer boundary ($x=r_{2}$). So, it is concluded that the same equilibrium saturation distribution is expected for both TEO and OEO setups, for same capillary pressure curves, and same rotational speeds.

Since both setups were acting similarly in their equilibrium state, it is expected that the Forbes methodology (Forbes, 1990) for the calculation of capillary pressure curves from centrifuge data is also applicable for the OEO setup. It should be called to mind that the Forbes approach is developed based on hydrostatic equilibrium state of fluids in centrifuge test, i.e., Eqs. (20) and (21).

**Figure 5:**
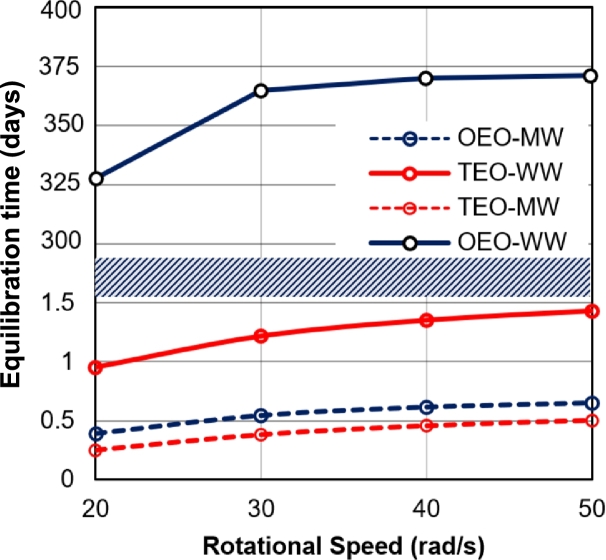
The equilibration time for TEO and OEO systems with both ww and mw capillary pressure curves. The shaded area is used for removing the empty space in the plot.

On the other hand, to ensure the feasibility of applying the new setup in practical situations, it is necessary to investigate the equilibration time in both systems. The equilibration time (that is defined in section 2.5) at each system is compared in Fig. 5. The centrifuge tests in TEO systems reached equilibrium at the time range of around 0.5 days in the mixed wet (*Pc_MW*) system and 1.4 days in the water-wet (*Pc_ww*) states. In the OEO system, although the equilibration process lasts 0.6 days for the *mw* case, it took around 360 days for the water-wet condition to meet the equilibrium conditions. It is clear that the time scale increases with water-wetness for both systems, however, it is more phenomenal in the OEO system. This time is especially significant at low rotational speeds where the mobility of a phase (here *nw* phase) is close to the endpoint (i.e., $\lambda_{nw}(r_{2}^{-},t)≅0$). The reason behind this observation is that when the measuring point is so close to the endpoint saturations, the flow will occur at low oil mobility values ($k_{ro}≅0$) and leads to a very low flow rate.

Overall, running the OEO tests for strongly wet conditions may be inefficient, especially when it is intended to find a capillary pressure curve. However, the timescale of the OEO test in a mixed-wet state is reasonable for conducting tests in a short time. This shows that the replacement of TEO tests and OEO tests for the calculation of capillary pressure curves in mixed wet conditions is technically and economically feasible in the case of necessity. More details about the impact of the wetting condition on the OEO test are provided in the following sections

### 3.3 Comparing the dynamic distribution of saturations

**Figure 6:**
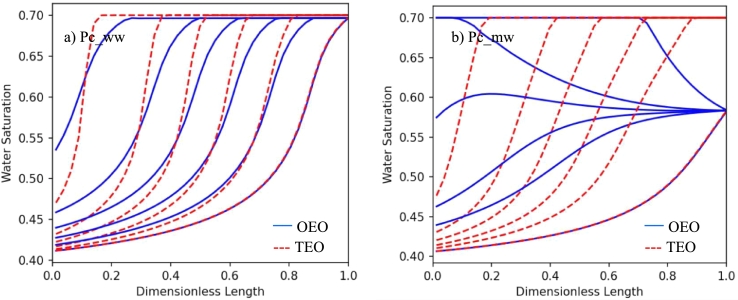
The dynamic saturation profile of the wetting phase before reaching an equilibrium in TEO and OEO systems for a rotational speed of 40 rad/s. In each case, the distributions are shown at recovery factors of 0.02, 0.10, 0.20, 0.30, and final recovery factors, from up to down, respectively. a) Water-wet capillary pressure curve, Pc_ww, b) Mixed-wet capillary pressure curve, Pc_mw.

As it is shown in the previous section, at identical rotational speeds, the fluid distributions in the core converge to similar equilibrium distributions in both TEO and OEO systems. In this section, it is tried to analyze the dynamic trend of converging to an equilibrium condition in two installations. Fig. 6 compares the dynamic saturation profile of the TEO and OEO systems for both setups where simulations are performed at the single rotational speed of 40 rad/s. The saturation distributions are exported at the same recovery factor fractions (i.e., 3%, 10%, 15%, 20%, 25%, and final recovery). As Fig. 6 shows, two setups show different dynamic behaviors, especially in the *mw* condition. The main reason is the different boundary conditions. In the OEO setup, $u_{T}=0$ condition is guaranteed on the $r_{2}$ boundary, while in TEO setup this limitation did not apply. This difference creates a different flow regime at the outer boundary.

**Figure 7:**
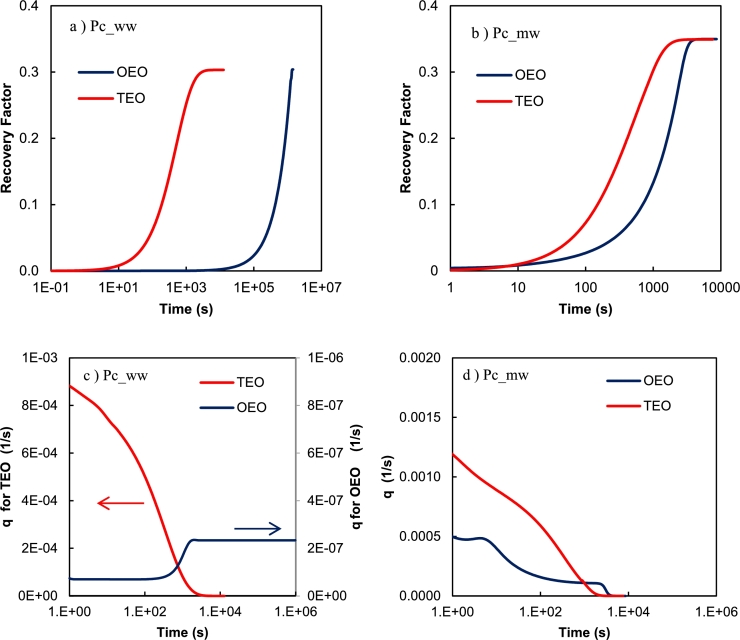
The recovery factor curve and recovery rate (*q* = ∂*RF*/∂*t*) versus time for TEO and OEO setups. a,b) Recovery factor curves in semilog scale for water-wet and mixed-wet capillary pressure curves, respectively; c,d) *q* vs. time for water-wet and mixed-wet capillary pressure curves, respectively.

In the TEO setup, for both *ww* and *mw* systems, the flow regime is co-current where the front moves from $x=r_{1}$ to $x=r_{2}$. In the OEO setup, oil enters the core at $x=r_{2}$ and the saturation at this face (the last cell) is reduced to reach the value corresponding to zero capillary pressure at beginning of the test. Then, the saturation profile moves toward the close boundary (from $r_{2}$ to $r_{1}$) and is finished with a saturation movement toward the open boundary (from $r_{1}$ to $r_{2}$). In this setup, the outer boundary ($x=r_{2}$) is a flowing gateway and acts as the bottleneck for flow in *ww* cases. It is because, in comparison to other points, $x=r_{2}$ has always highest $S_{w}$ (saturation corresponding to the zero P_c_) and the lowest $\lambda_{nw}$. So, the test time in this setup is directly controlled by the $S_{w}$ at $x=r_{2}$. This point is more lucid in Fig. 7. This figure shows the recovery curve for both TEO and OEO setups under two wetting conditions. It is clear that the test time in the *ww* condition is significantly influenced by the boundary conditions, while in the *mw* condition the difference is lower. The recovery rate (q=∂RF/∂t1/s) of the two setups is compared in Figs. 7c, and d. As it is clear, the rate in the TEO setup is higher in the early and intermediate times of production. This difference is more significant in the *ww* condition. This is a good indication that in contradiction to the TEO setup, the OEO case has lower changes in production rate over time. This behavior can be effective in the easier recording of produced volume during the test. The reason that the production rate for the OEO case in Fig. 7c stays constant is that in this setup the bottleneck of the flow is the saturation at the $r_{2}$ boundary. So, after the time that the saturation at that point reaches an equilibrium, the rate will be constant for a long time. It should be considered that the difference between the production rate in the two curves is 3 orders of magnitude.

### 3.4 Threshold capillary pressure

**Figure 8:**
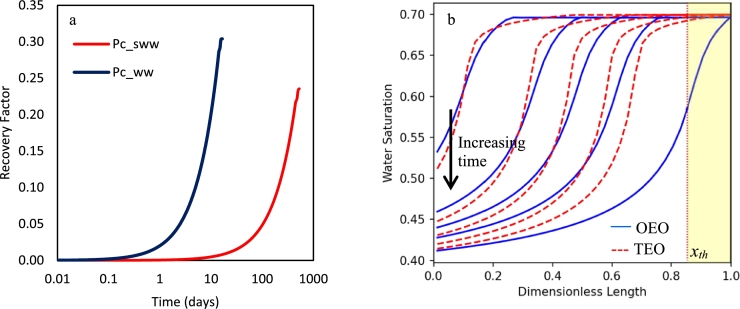
Comparison of the OEO centrifuge setups for cases with different capillary threshold values, i.e., *P*_*c*__*ww* and *P*_*c*__*sww*. (a) Recovery curve in semilog scale, (b) saturation profile curves are shown for the equal recovery factors of 3%, 10%, 15%, 20%, and final recovery factor.

The threshold capillary pressure ($P_{c}^{th}$), or entry capillary pressure, is the ability of a porous media saturated with a wetting phase in blocking the flow of the *nw* phase. The value corresponds to the capillary pressure that is acting due to the largest pores in the porous media and shows itself as a resistive force in drainage processes. In this section, it is aimed to see how the value of $P_{c}^{th}$ affects the flow profile in the OEO centrifuge test. To do that, the OEO setup is spun with two separate capillary pressure curves of Pc_ww and *Pc_sww* (see Fig. 3), while other properties, including *ω* (=40 rad/s) assumed constant (see Table 1). The threshold pressure in *Pc_ww* is zero, while in *Pc_sww*, it is equal to 0.01 bar. As Fig. 8a shows, the presence of $P_{c}^{th}$ significantly increases the test time. In this case, the equilibrium time (for a single rotational speed) was 13 days for the $P_{c}\_ww$ curve, while it was close to 500 days for the $P_{c}\_sww$ curve.

This noticeable rise in time is interpreted by the low *nw* mobility region that is established inside the core, from $x_{th}$ (threshold equivalent length) to $r_{2}$ boundary. The location of $x_{th}$ (Eq. (25)) is correlated to the value of $P_{c}^{th}$ and is calculated by replacement of $P_{c}^{th}$ in Eq. (20) (Andersen et al., 2020).

(25) $$x_{th}=\sqrt{r_{2}^{2}-\frac{2P_{c}^{th}}{\Delta \rho \omega^{2}}},$$

where the length of this region is $r_{2}-x_{th}$. In our case, this length is 0.7 cm, or in dimensionless unit $r_{2}-x_{th}=0.14$, as highlighted (yellow) in Fig. 8*b*. This figure also compares the saturation profiles in two cases (related to the equal recovery factors of 3%, 10%, 15%, 20%, and final recovery factor), where the value of $x_{th}$ had significant effects on the final distribution of phases. Overall, the timescale of tests in high $P_{c}^{th}$ conditions seem to be unfeasible. So, in this condition, an OEO setup is not recommended.

### 3.5 Viscosity ratio

Flow in porous media is highly under influence of the viscosity ratio ($M=\mu_{o}/\mu_{w}$) of invading and defending phases (Haugen et al., 2015). In this section, it is intended to compare the variations in the results of TEO and OEO centrifuge capillary pressure setups with changes in viscosity ratios. The simulations are carried out at a single rotational speed of 40 rad/s (starting from 0 rad/s). The water-wet and mixed wet capillary pressure curves (*Pc_ww*) are used separately for each case.

**Figure 9:**
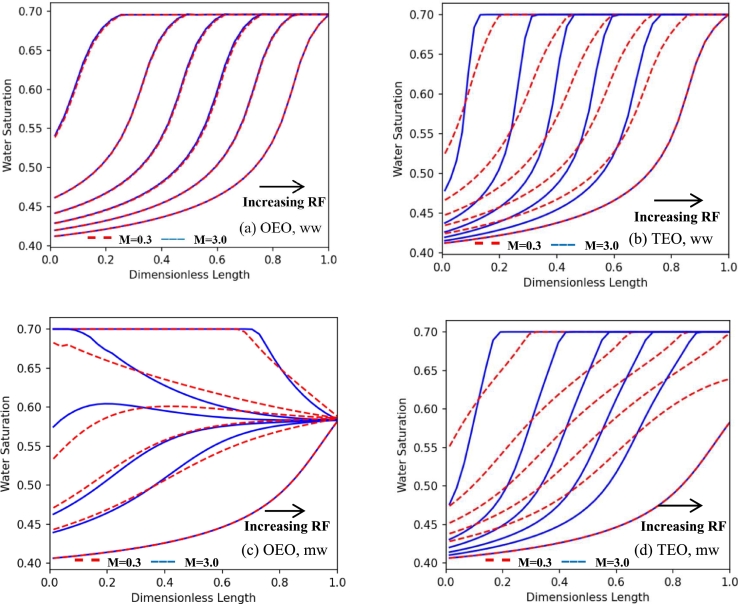
The dynamic trend of saturation profile at different times for different viscosity ratios, installations, and wetting conditions. For all cases, blue line: *μ*_*o*_/*μ*_*w*_ = 3, red dash: *μ*_*o*_/*μ*_*w*_ = 0.3. (a) OEO, ww; (b) TEO, ww; (c) OEO, mw; (d) TEO, mw. All the results are related to the centrifuge drainage test with the single rotational speed of 40 rad/s and the distributions are extracted for the recovery factors of 3%, 10%, 15%, 20%, 25%, and final recovery factor.

Fig. 9 shows the changes in the distribution of saturations before equilibration for both TEO and OEO setups and oil to water viscosity ratios ($M=\mu_{o}/\mu_{w})$ of 3 and 0.30, where the water viscosity was kept constant (0.7 cP) and two oil viscosities of 2.1 and 0.21 cP were used. The distributions are shown at similar fractions of water recovery factors (3%, 10%, 15%, 20%, 25%, and final recovery factor). As Fig. 9 shows, reducing the viscosity ratio from 3 to 0.3 resulted in same equilibrium saturation profiles for both OEO and TEO boundary conditions. This is an indication that the calculated capillary pressure curves in the centrifuge system are not dependent on the viscosity ratio of fluids (in agreement with Eq. (21)). However, the results show that their dynamic behavior is altered by the viscosity ratio. In the TEO setup, the dynamic saturation profiles can be influenced by the viscosity ratio during the transition from the previous equilibrium state to the new state (Figs. 9 b and d). In this setup, increasing the viscosity ratio improved the frontal behavior of the system. This conclusion is true for both wetting conditions. On the other hand, as Fig. 9a shows, the flow regime in the OEO setup is not influenced by the changes in viscosity ratio in water-wet conditions, although the equilibration timescale is highly increased. However, in the mixed-wet condition, there are some small changes in the saturation profile, as shown in Fig. 9c.

**Figure 10:**
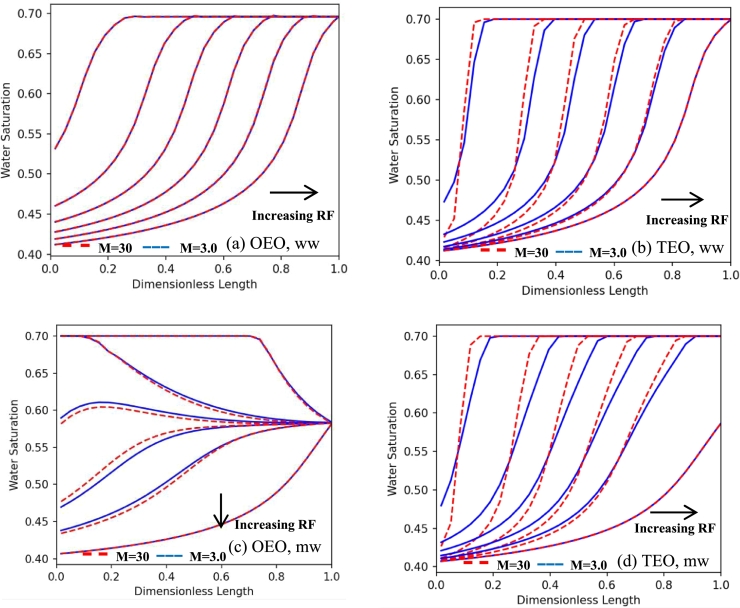
The dynamic trend of saturation profile at different times for different viscosity ratios, installations, and wetting conditions. For all cases, blue line: *μ*_*o*_/*μ*_*w*_ = 3, red dash: *μ*_*o*_/*μ*_*w*_ = 30, where oil viscosity is kept constant (2.1 cp) and water viscosities are 0.7 and 0.07 cp, respectively. (a) OEO, ww; (b) TEO, ww; (c) OEO, mw; (d) TEO, mw. All the results are related to the centrifuge drainage test with the single rotational speed of 40 rad/s and the distributions are extracted for the recovery factors of 3%, 10%, 15%, 20%, 25%, and final recovery factor.

In another case, it is tried to change the water viscosity. So, previous simulations are performed with oil to water viscosity ratios ($M=\mu_{nw}/\mu_{w})$ of 3 and 30, where the oil viscosity is kept constant (2.1 cP) and two water viscosities of 0.7 and 0.07 cP were used. As it is clear from Fig. 10, the trends are almost the same as in the previous case, although the sensitivity of saturation profiles to viscosity ratio was lower. So, it can be concluded that changes in oil viscosity lead in more changes in comparison to water viscosity.

### 3.6 Calculation of relative permeability

Relative permeability is known as one of the most determining parameters in fluid flow in porous media. One of the applications of centrifuge capillary pressure tests is the inverse calculation of relative permeability curves by history matching of the recovery profile. However, since the measurement of the production profile is always mixed with experimental errors, analyzing the likely uncertainties in the calculated curves is vital. In this section, it is planned to investigate the possibility of calculation of relative permeability by inversed history matching of centrifuge test production profile. To do that, for a known simulation case, the production profile was recorded, and the recorded profile was used as the observed (experimental) data for the history matching task. In history matching, it was assumed that all parameters of flow are known, and only relative permeability variables are needed to be determined. Since finding the relative permeability parameters needs an iterative trial of simulations with different relative permeability realizations, the look-up process for the best values was managed by a PSO algorithm and a wide range of parameters (Table A2). Full details about the approach are provided in Appendix C.

Before investigating the results of history matching, a sensitivity analysis on the impact of different relative permeability parameters (when the values were increased by 20%) on the recovery curve of TEO and OEO setups was performed. Table 3 shows the final results where the full details of the simulations, including recovery curves, can be found in Appendix C.1. As it is clear that for this case, the sensitivity to the *nw* phase parameters ($k_{nw}^{⁎}$, $n_{nw}$) is more significant than *w* phase-related variables. This can be an indication that the *w* phase-related variables are more prone to measurement errors. Also, on average, OEO was more sensitive to the deviations in comparison to the TEO setup (especially for the *nw* phase) which can be an indication of the lower probability of calculation of relative permeability with the OEO setup.

**Table 3:** The dependency of recovery profiles to the changes (20%) in Corey parameters for TEO and OEO cases. More details are provided in Appendix C.1.

Corey parameter	Mean Squared Error (MSE)		
	TEO	OEO	Average
$k_{w}^{⁎}$	0.011	0.012	**0.011**
$k_{nw}^{⁎}$	0.009	0.057	**0.033**
*n*_*w*_	0.001	0.006	**0.003**
*n*_*nw*_	0.032	0.123	**0.061**
Average	**0.013**	**0.049**	**0.027**

In the following, the results of history matching are discussed, while the full details including figures are provided in Appendix C.2. The summary of the history matching results is shown in Table 4. Three different experimental procedures are investigated here:

1. **TEO:** The centrifuge was spun only with a TEO setup.
2. **OEO:** The centrifuge was spun only with an OEO setup.
3. **TEO & OEO:** The centrifuge is spun for both TEO and OEO setups. So, the history matching

is carried out for both setups at the same time. The average error for each setup is considered as the loss function

**Table 4:** The results of history matching of production profile for single optimization of TEO and OEO setups, and simultaneous optimization of both setups.

Case	Optimized variables				Mean Square Error		
	$k_{w}^{⁎}$	$k_{nw}^{⁎}$	*n*_*w*_	*n*_*nw*_	*k*_*w*_	*k*_*nw*_	total
TEO	0.15	0.36	0.89	1.82	9.7e-05	1.7e-05	5.7e-05
OEO	0.15	0.37	0.92	1.84	8.1e-05	6.7e-05	7.4e-05
TEO & OEO	0.15	0.37	0.88	1.83	1.1e-04	7.4e-05	9.4e-05

**Figure A6:**
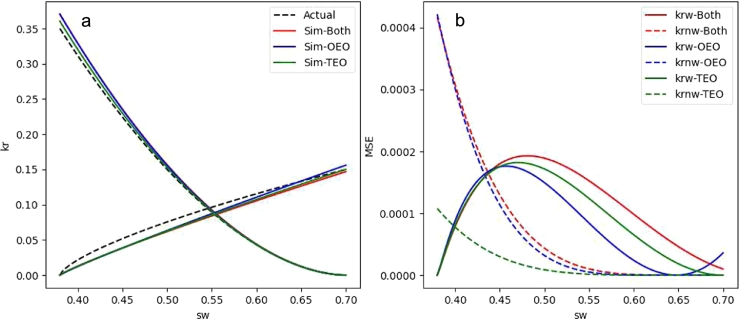
The calculated relative permeability curves by history matching of production profile, based on fitting of TEO, OEO, and both setups. (a) The fitted relative permeability curves, and (b) the MSE error (Eq. (47)) between actual and simulation relative permeability curves vs. *s*_*w*_ for all cases.

These results were obtained after 800 unique simulation runs (for each setup) with different relative permeability curves, guided by the Particle Swarms Optimization (PSO) algorithm. As it is clear from the obtained results, in total, the accuracy of the predictions for all 3 cases is reasonable. A graphical presentation of the calculated curves and their Mean Square Error (MSE), Eq. (47), vs. $s_{w}$ is shown in Fig. A6.

To look deeper into the sensitivity of the obtained results to different parameters, the whole optimization task was repeated for cases with different viscosity ratios. The viscosity values, as well as the results, are summarized in Table 5 (the provided MSE values are for optimization of the OEO setup). From the results, it can be concluded that the MSE in the calculated relative permeability is more for the phase with lower viscosity (lower flow resistances). So, it can be expected that the overall uncertainties of the calculated relative permeability curves are much higher in problems with a viscosity ratio far from 1, i.e., M≪1 or M≫1.

**Table 5:** The impact of viscosity ratio on the uncertainty in the calculated relative permeability (for OEO setup).

Viscosity (cp)		M	MSE		
*μ*_*w*_	*μ*_*nw*_	*μ*_*nw*_/*μ*_*w*_	*k*_*w*_	*k*_*nw*_	total
0.71	2.71	3.85	8.1e-05	6.7e-05	7.4e-05
0.07	2.71	38.57	5.8e-04	2.7e-05	3.0e-04
0.71	0.27	0.38	1.7e-04	2.0e-03	1.1e-03

## 4 Discussion

In this work, a new installation for the centrifuge capillary pressure test labeled OEO was introduced and compared to the classic installation (TEO). Afterward, the behavior of these setups under different conditions was investigated and compared together. We showed that both systems provide similar results in the calculation of capillary pressure curves.

However, there were found some major differences in the dynamic transition of saturation profile from an equilibrium state to another one (with the same relative permeability curves). The main difference between the two systems is the flow regime of the phases, where in the TEO system the fluids flow co-currently, while in the OEO system, the dominant flow regime is counter current. Considering these differences, the OEO installation is proposed for the calculation of counter-current relative permeabilities, which is essential in many disciplines such as migration of CO_2_ in saline aquifers (Javaheri and Jessen, 2011), and hydrocarbon production from naturally fractured reservoirs during secondary/tertiary recovery operations (Aronofsky et al., 1958).

Here, the recommended technique for calculation of the relative permeability was trial-based history matching of the production profile. This technique could give a reasonable prediction of the relative permeability curve in hypothetical scenarios discussed in previous sections. The method was also recommended in the work of Mahzari et al. (2018) for core flooding tests. Sylte et al. (2004) concluded that the calculated relative permeabilities are more trustable if the flow occurs in a wide variety of saturation ranges. In centrifuge capillary pressure tests, the correct measurement of produced volume is a challenge in many cases. This measurement error may lead to uncertainty in the calculated relative permeability curves. However, one of the specifications of the OEO setup is its production rate which is almost constant during the test (see Fig. 7). So, the recovery curve in the cartesian scale has a linear trend. This makes the measurement of the produced volume easier since it can be obtained by only a few points. By the way, the automated setups for high-accuracy and high-frequency data gathering from centrifuge test (Ferno et al., 2007) are recommended in both installations for lowering the possible uncertainties in the calculated relative permeabilities. On the other hand, one of the main drawbacks of the centrifuge capillary pressure test (in classic TEO setup), as discussed by McPhee et al. (2015), is that at least at the earliest time of changing the rotational speed, a significant driving force is applied to the rock and fluids that lead to a high de-saturation rate which is much higher than the actual values we see in nature. This may change the flow regime behavior of fluids. More importantly, this force may lead to rock failure and disintegration especially in rocks with low geomechanical strengths. Considering the results shown in Fig. 7, since the maximum flow rate (at the beginning minutes of the test) is lower in the OEO setup (in comparison to the TEO setup), it possibly reduces the flowing stresses applied to the rock grains, that likelihood, the chance of rock failure is decreased. This is a great advantage in unconsolidated rocks where they usually collapse at high rotational speeds. It should be reminded that the most extreme fluxes are applied to the system at the beginning seconds of flow when the rotations just started and by passing time and reducing the flow rate, the applied stresses vanish slowly. However, further analysis of this theory needs geomechanical studies that are out of the scope of this paper.

In the *mw* state, the time for both TEO and OEO setups is acceptable for both calculations of capillary pressure and relative permeability. In the *ww* state, the timescale of the OEO test is significant. So, in this wetting state running this test may be unfeasible. To save the testing time, the TEO setup can previously be launched to obtain the capillary pressure curve. It is important to note that if the capillary pressure curve is calculated in the TEO system, the OEO test can be launched with fewer numbers of rotational steps, so the test time/cost would be significantly reduced. Furthermore, since we showed that OEO setups are not sensitive to the viscosity values (i.e., viscosity of the phase with the lowest mobility), the OEO setup may also be run with fluids with lower viscosities. We should also bear in mind that always there are methods to estimate or determine the wetting state of the system, such as contact angle test, and spontaneous imbibition test.

Finally, it is decent to mention that although the obtained results in this work demonstrated the applicability of using the OEO setup for calculation of both capillary pressure, and relative permeability curves, however, it is still advantageous to experimentally verify the model and the related parameters in the future. Furthermore, the analysis of the features of the new setup in the imbibition flow processes is recommended in future studies.

## 5 Conclusions

In this work, a numerical model for simulation of centrifugal capillary pressure test in two different boundary conditions (one end open, and two ends open) is provided. The developed model for the TEO setup was validated against the Sendra v2018.5 simulation software. Below points are concluded in this work:

• The equilibrium saturation profiles for both TEO and OEO installations follow the same distributions. So, similar capillary pressure curves can be obtained in both installations.
• The dynamic trend of flow and distribution of fluids are different in the two setups. This difference is due to the different boundary conditions, where the TEO setup creates a co-current flow regime, while it is counter-current in the OEO setup.
• The equilibration time (i.e., test time) for the OEO system is higher than the time for the TEO system, especially in more water-wet cases. This is due to the definition of boundary conditions where the *nw* phase has very low mobility at points close to the outer boundary ($r_{2}$). However, the time scale of the test for the two cases is comparable and acceptable in mixed-wet rocks.
• In the water-wet capillary pressure state, the dynamic saturation profile in the TEO setup is significantly influenced by the viscosity ratio of fluids, and more viscosity ratio created more frontal flow behavior. The impacts of *nw* viscosity (the invading phase) in changing the flow regime were more significant in comparison to the *w* phase.
• In the OEO setup, no sensitivities to the viscosity ratio (in either viscosity) were found in the water-wet system, however, a slight change in the dynamic saturation profile is observed at mixed-wet conditions.
• The equilibration time of the flow is significantly influenced by the viscosity values that may lead to changes in the economic feasibility of centrifuge tests, especially in the OEO system. The time scale was more dependent on the viscosity of the most mobile phase.
• The presence of the threshold capillary pressure significantly rises the test time for the OEO setup by establishing a low *nw* mobility region close to the open boundary, that limits the flowing velocity of fluids.
• Considering the long temporal duration of performing the OEO centrifuge test in cores with water wet behavior, running OEO tests may be infeasible in these cores, especially in cases with high threshold capillary pressures. However, since we showed that the OEO model is almost insensitive to the viscosity of fluids, the OEO test can be run with different viscosity values. This approach can be used for reducing the test time of OEO setups.
• This newly proposed setup should help to calculate the counter-current relative permeability by an inverse calculation method with reasonable accuracy and applicability in real conditions. It can be performed by history matching the production volume time series. Our investigations regarding the OEO setup showed that obtaining a unique relative permeability is guaranteed under different conditions.
• The findings reported here also shed new light on the uncertainties in the inverse calculation of relative permeability by centrifuge setup. For the OEO setup, it was found that in the viscosity ratios far from 1 (M≪1 or M≫1), the relative permeability corresponding to the lower viscosity phase may show a higher level of errors.

**Nomenclature**

**Table:** 

**Roman**			
*a*,*b*	=	Capillary pressure correlation parameters,	Pa
*k*	=	Capillary pressure correlation parameter,	–
*K*	=	Absolute permeability,	m^2^
$k_{w}^{⁎}$	=	Endpoint wetting phase relative permeability,	–
$k_{nw}^{⁎}$	=	Endpoint non-wetting phase relative permeability,	–
*k*_*ri*_	=	Relative permeability,	–
*n*_*nw*_,*n*_*w*_	=	Corey exponents,	–
*p*_*c*_	=	Capillary pressure,	Pa
*p*_*i*_	=	Phase pressure,	Pa
*r*_1_	=	Inner radius,	m
*r*_2_	=	Outer radius,	m
*s*_*w*_	=	Water saturation,	–
*s*_*wc*_	=	Connate water saturation,	–
*u*_*i*_	=	Darcy phase velocity,	m/s
*u*_*T*_	=	Darcy total velocity, m/s	
*x*_*th*_	=	Equivalent threshold length,	m
*q*	=	Recovery rate,	1/s

**Greek**			
Δ*ρ*	=	Density difference,	kg/m^3^
*λ*_*i*_	=	Phase mobility,	1/(Pa s)
*λ*_*T*_	=	Total mobility,	1/(Pa s)
*μ*_*i*_	=	Phase viscosity,	Pa s
*ρ*_*i*_	=	Phase density,	kg/m^3^
*σ*_*ow*_	=	Interfacial tension,	N/m
*ϕ*	=	Porosity,	-
*ω*	=	Rotational speed,	rad / s

**Indices**			
*eq*	=	Equilibrium state of a cycle	
*i*	=	Phase index	
*nw*	=	Non-wetting phase	
*w*	=	Wetting phase	

## Declarations

### Author contribution statement

**Jassem Abbasi:** Performed the experiments; Analyzed and interpreted the data; Contributed reagents, materials, analysis tools or data; Wrote the paper. **Pal Andersen:** Conceived and designed the experiments; Analyzed and interpreted the data; Contributed reagents, materials, analysis tools or data.

### Funding statement

This research did not receive any specific grant from funding agencies in the public, commercial, or not-for-profit sectors.

### Data availability statement

Data associated with this study has been deposited at https://github.com/jcabbasi/centrifuge_public.

### Declaration of interests statement

The authors declare no conflict of interest.

### Additional information

No additional information is available for this paper.

## Figures and Tables

**Table:** 

*Algorithm 1: pseudo-code for solving the flow equations*.	
**Input**: rock & fluid properties, saturation functions, setup settings	
**Output**: $S_{w}$ & *P vs. time*	
**Ensure**: Eqs. (38), (39) are satisfied	

1	**for***ω*:
2	**while***production*not*stopped*:
3	calculate Δ*t*
4	**while**not*converged*
5	calculate *F*_*i*1_*for cells.*
6	calculate *F*_*i*2_*for cells.*
7	calculate *derivatives of F*_*i*1_*and F*_*i*2_*vs. s*_*w*_*and p*
8	generate solution matrix: *A and b in AX* = *b*
9	calculate *the inverse of A matrix*
10	find Δ*s*_*w*_*and* Δ*p by X* = *A*^−1^*b*
11	$s_{w}^{n+1}\leftarrow s_{w}^{n}+\Delta s_{w}$
12	*p*^*n*+1^ ← *p*^*n*^ + Δ*p*

**Figure A1:**
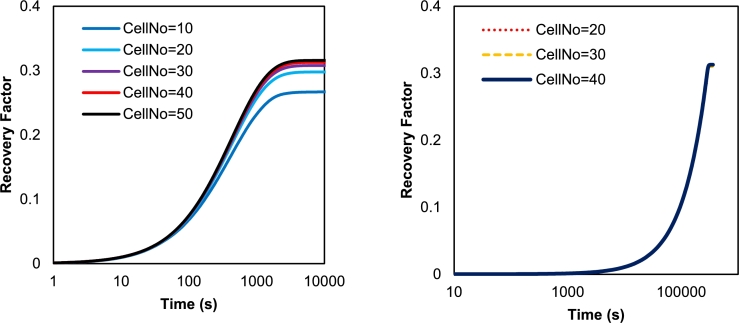
Sensitivity Analysis on the grid numbers for minimization of the numerical dispersion errors: TEO case in semilog scale (left), OEO case in semilog scale (right).

**Figure A2:**
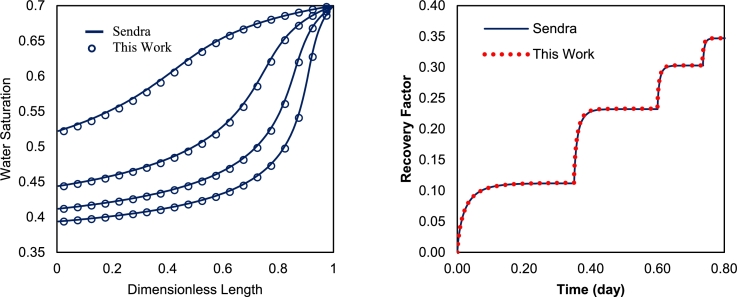
Comparison of the model developed in this work and the results of Sendra v2018.5 for the TEO system, for the rotational speeds of 20, 30, 40, and 50 rad/s. Comparison of simulated saturation profiles through the core (left). The line represents simulations from Sendra v2018.5, and markers show the results of the numerical model developed in this work. The comparison of recovery factor versus time for all rotational speeds (right).

**Figure A3:**
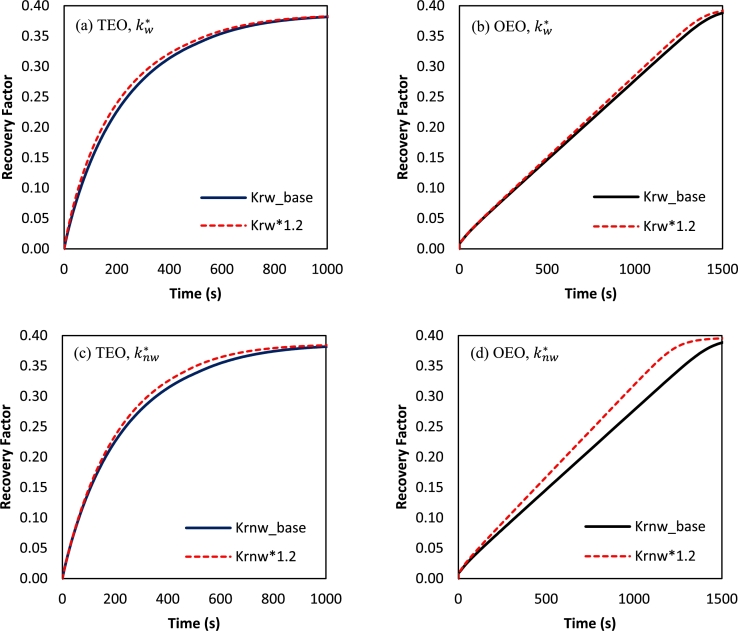
The sensitivity analysis on the impacts of 20% changes in $k_{w}^{⁎}$ and $k_{nw}^{⁎}$ on production profile for both OEO and TEO cases. (a) $k_{w}^{⁎}$, TEO; (b) $k_{w}^{⁎}$, OEO; (c) $k_{nw}^{⁎}$, TEO; (d) $k_{nw}^{⁎}$, OEO.

**Figure A4:**
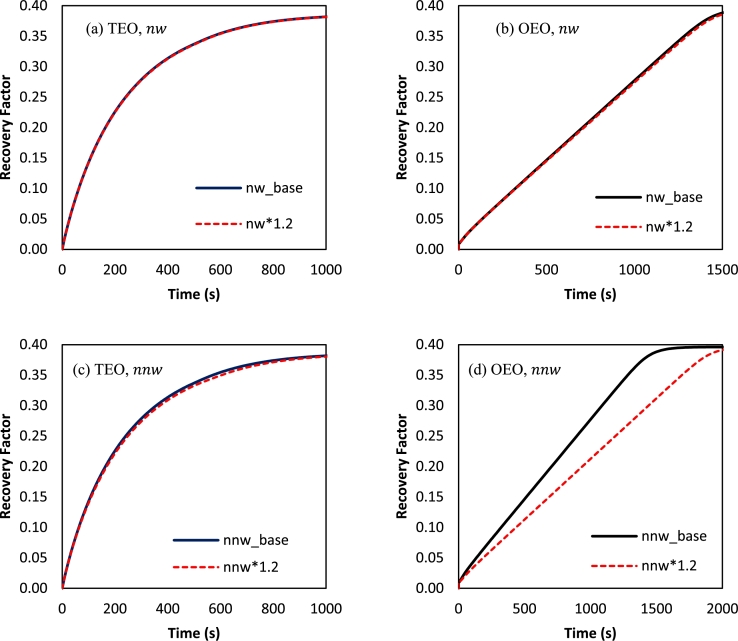
The sensitivity analysis on the impacts of 20% changes in Corey Equation parameters on production profile for both OEO and TEO cases. (a) *n*_*w*_, TEO; (b) *n*_*w*_, OEO; (c) *n*_*nw*_, TEO; (d) *n*_*nw*_, OEO.

**Figure A5:**
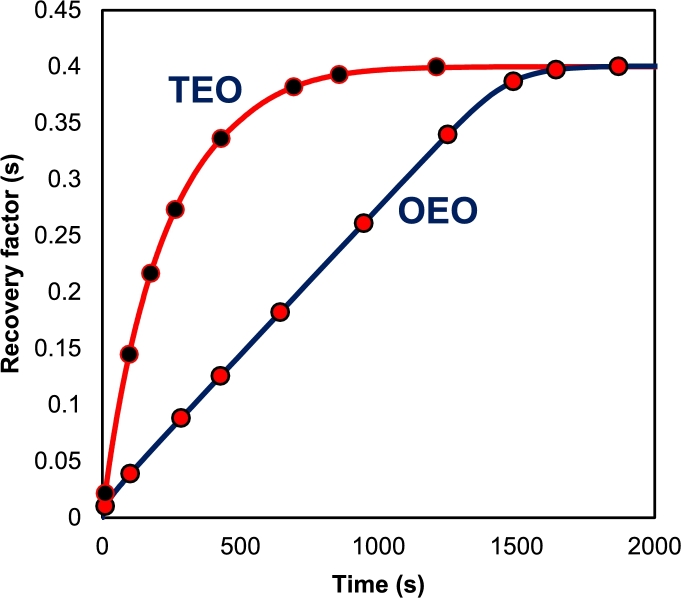
The production curve, used as the observation (experimental) data for inverse calculation of the relative permeability. The line is the original production data, and the points are the selected points for optimization.

**Table A1:** The meta parameters of the PSO algorithm.

Properties	Value	Properties	Value
Number of dimensions*	4	Run No	800
Number of particles	40	$c_{\text{1}}$	0.7
Objective function	MSE	$c_{\text{2}}$	0.7

* Number of optimization variables.

**Table A2:** The range of variables was fed to the PSO algorithm for recovery profile optimization.

Variable	*n*_*w*_	*n*_*nw*_	$k_{w}^{⁎}$	$k_{nw}^{⁎}$
Actual	*0.7*	*1.8*	*0.15*	*0.35*
Minimum	0.1	0.1	0.1	0.1
Maximum	5	5	1	1

**Figure A7:**
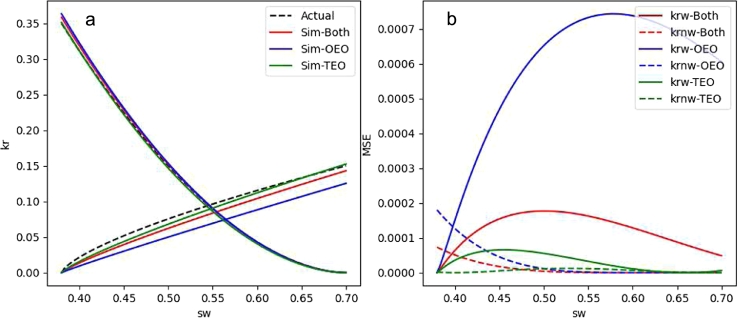
The calculated relative permeability curves based on fitting of TEO, OEO, and both setups for the cases with very low w phase viscosity (*μ*_*w*_ = 0.07 cp). (a) The fitted relative permeability curves and the actual curves (b) the MSE (Eq. (47)) error between actual and simulation curves vs. *s*_*w*_ for all cases.

**Figure A8:**
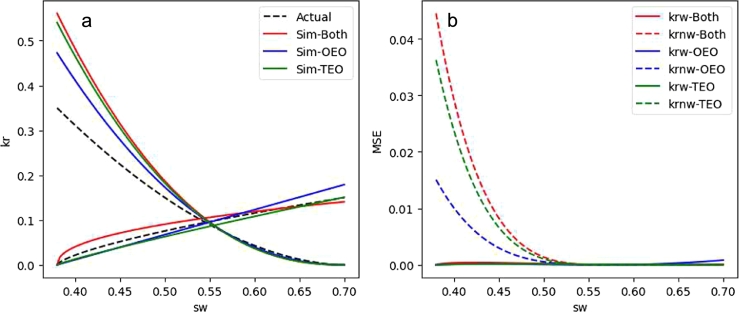
The result of history matching of production profile, based on fitting of both TEO and OEO setups for the case with very low nw phase viscosity (*μ*_*nw*_ = 0.217 cp). (a) The fitted relative permeability curves, and (b) the MSE (Eq. (47)) error between actual and simulation curves for vs. *s*_*w*_ for all cases.

## Appendix A Model discretization and implicit solution

A fully implicit, i.e., implicit pressure and implicit saturation, scheme was employed to solve the equations, which is outlined in the following. The original Eqs. (1), (2), and (3) can be expressed using $s_{w}$ and $p_{w}$ as the unknown variables after the elimination of $s_{o}$ and $p_{o}$. This is done by inserting the flux relations into the mass balances and then replacing the *nw* phase equation with the sum of the two mass balances.

(26) $$\phi \partial_{t}(s_{w})-\partial_{x}(\lambda_{w}[\partial_{x}p_{w}-\rho_{w}\omega^{2}x])=0$$

(27) $$\partial_{x}(\lambda_{nw}\partial_{x}P_{c}+\lambda_{T}\partial_{x}p_{w}-\omega^{2}x(\lambda_{nw}\rho_{nw}+\lambda_{w}\rho_{w}))=0$$

We then discretize the equations. In the following, we let wetting phase pressure and saturation be denoted *p* and *s* respectively, and the indices *i* and *n* refer to grid cell and time step, respectively.

(28) $$s_{i}^{n+1}-s_{i}^{n}-\frac{1}{\phi }\frac{\Delta t}{\Delta x}[\lambda_{w,i+\frac{1}{2}}^{n+1}(\frac{p_{i+1}^{n+1}-p_{i}^{n+1}}{\Delta x}-\rho_{w}\omega^{2}x_{i+\frac{1}{2}})-\lambda_{w,i-\frac{1}{2}}^{n+1}(\frac{p_{i}^{n+1}-p_{i-1}^{n+1}}{\Delta x}-\rho_{w}\omega^{2}x_{i-\frac{1}{2}})]=0$$

(29) $$\lambda_{nw,i+\frac{1}{2}}^{n+1}\frac{P_{c,i+1}^{n+1}-P_{c,i}^{n+1}}{\Delta x}+\lambda_{T,i+\frac{1}{2}}^{n+1}\frac{p_{i+1}^{n+1}-p_{i}^{n+1}}{\Delta x}\;\;-\omega^{2}x_{i+\frac{1}{2}}(\lambda_{nw,i+\frac{1}{2}}^{n+1}\rho_{nw}+\lambda_{w,i+\frac{1}{2}}^{n+1}\rho_{w})\;-\lambda_{nw,i-\frac{1}{2}}^{n+1}\frac{P_{c,i}^{n+1}-P_{c,i-1}^{n+1}}{\Delta x}-\lambda_{T,i-\frac{1}{2}}^{n+1}\frac{p_{i}^{n+1}-p_{i-1}^{n+1}}{\Delta x}\;\;+\omega^{2}x_{i-\frac{1}{2}}(\lambda_{nw,i-\frac{1}{2}}^{n+1}\rho_{nw}+\lambda_{w,i-\frac{1}{2}}^{n+1}\rho_{w})=0$$

We make the following evaluations of the boundary and functional terms:

(30) $$\lambda_{k,i+\frac{1}{2}}^{n+1}=\frac{1}{2}[\lambda_{k}(s_{i}^{n+1})+\lambda_{k}(s_{i+1}^{n+1})],\;(k=n,nw,T)$$

(31) $$\lambda_{k,i-\frac{1}{2}}^{n+1}=\frac{1}{2}[\lambda_{k}(s_{i-1}^{n+1})+\lambda_{k}(s_{i}^{n+1})],\;(k=n,nw,T)$$

(32) $$x_{i+\frac{1}{2}}=\frac{1}{2}[x_{i}+x_{i+1}],\;x_{i-\frac{1}{2}}=\frac{1}{2}[x_{i-1}+x_{i}]$$

(33) $$P_{c,i+1}^{n+1}=P_{c}(s_{i+1}^{n+1}),\;P_{c,i}^{n+1}=P_{c}(s_{i}^{n+1}),\;P_{c,i-1}^{n+1}=P_{c}(s_{i-1}^{n+1})$$

Eqs. (28) and (29) for cell *i* depend on six unknowns, namely the water saturation and the water pressure in cell *i* and its two neighbor cells: $s_{i-1}^{n+1},p_{i-1}^{n+1},s_{i}^{n+1},p_{i}^{n+1},s_{i+1}^{n+1},p_{i+1}^{n+1}$. The mobilities and geometrical vectors (x) are discritized centrally (Eqs. (30), and (31)). Capillary pressure values also solved implicitly (Eq. (33)).

The boundary cells require special treatment and depend on the boundary conditions used (TEO or OEO). Ghost cells are used to implement the boundary conditions. In both cases the outermost ghost cell $\left(i=N_{x}+1\right)$ has a defined zero water and capillary pressure. However, the *nw* phase cannot leave at this side in both cases and is assigned zero mobility across the interface in the TEO case (in the OEO setup, it can flow toward inside the core). The conditions used in this cell thus become same as Eq. (34).

(34) $$p_{N_{x}+1}^{n+1}=0,\;s_{N_{x}+1}^{n+1}=1-s_{nwr}.$$

In the innermost cell, the conditions depend on whether the boundary is open or closed. Assume first the boundary is open to oil (TEO). The oil pressure is defined, but the water pressure is not continuous. Referring to the Eqs. (9) and (11), we can write:

(35) $$s_{0}^{n+1}=s_{wr},\;\lambda_{w,\frac{1}{2}}^{n+1}=0,\;(\text{TEO})$$

Eq. (35) indicates that water cannot enter/leave the core from the inner side. This assures that only oil has the mobility to flow across the boundary. We must make sure that the remaining boundary conditions define the correct oil pressure gradient. This is done by setting the capillary pressure and water pressure parameters in the innermost ghost cell such that:

(36) $$P_{c,0}^{n+1}+p_{0}^{n+1}=p_{o,0}^{n+1}=-\frac{1}{2}\rho_{nw}\omega^{2}(r_{2}^{2}-r_{1}^{2}),\;(\text{TEO})$$

To obey boundary conditions related to Eqs. (12) and (36) note that the individual values of $P_{c,0}^{n+1}$ and $p_{0}^{n+1}$ do not matter, as it is their sum (defining the oil pressure) that plays a role. In the OEO case (closed inner boundary) the flux of both phases is zero at the innermost boundary. Following the Eqs. (14), (16) and (17), this is set by defining all mobilities to zero at the inner boundary:

(37) $$\lambda_{k,\frac{1}{2}}^{n+1}=0,\;(k=n,nw,T),\;(\text{OEO}).$$

For simplicity, the values of $p_{c,0}^{n+1},p_{0}^{n+1}$ and $s_{0}^{n+1}$ are set as in the TEO case, but do not play a role in this case. It can be seen that the boundary condition shown by Eq. (15), forces the total velocity to be zero.

According to the above description, we get two discretized equations of the same form for each cell, which depend on the saturation and pressure at the next time step of that cell and its two neighbors. For the boundary cells, one of the neighbor cells is a ghost cell with constant values of saturation, water pressure, and capillary pressure. The equations can be written in the form:

(38) $$F_{i1}(s_{i-1}^{n+1},p_{i-1}^{n+1},s_{i}^{n+1},p_{i}^{n+1},s_{i+1}^{n+1},p_{i+1}^{n+1})=0,\;(i=1:N_{x})$$

(39) $$F_{i2}(s_{i-1}^{n+1},p_{i-1}^{n+1},s_{i}^{n+1},p_{i}^{n+1},s_{i+1}^{n+1},p_{i+1}^{n+1})=0,\;(i=1:N_{x})$$

The expressions $F_{i1}$ and $F_{i2}$ correspond exactly to the left-hand side of Eqs (41) and (42). The equations are strongly nonlinear and will be solved using Newton-Raphson iterations to obtain the solution at the new time step.

Assume that the solution in terms of pressures and saturations is known at timestep *n* and that the solution at the next time is going to be estimated by solving Eqs. (26) and (27). To solve these nonlinear equations, the equations are linearized as Eqs. (40) to (42) and iterated to find the solution at the new time step.

(40) $${\left[\frac{\partial F_{i1}}{\partial s_{i-1}}\right]}^{k}\Delta s_{i-1}^{k}+{\left[\frac{\partial F_{i1}}{\partial p_{i-1}}\right]}^{k}\Delta p_{i-1}^{k}+{\left[\frac{\partial F_{i1}}{\partial s_{i}}\right]}^{k}\Delta s_{i}^{k}+{\left[\frac{\partial F_{i1}}{\partial p_{i}}\right]}^{k}\Delta p_{i}^{k}\;\;+{\left[\frac{\partial F_{i1}}{\partial s_{i+1}}\right]}^{k}\Delta s_{i+1}^{k}+{\left[\frac{\partial F_{i1}}{\partial p_{i+1}}\right]}^{k}\Delta p_{i+1}^{k}+F_{i1}^{k}=0,{\left[\frac{\partial F_{i2}}{\partial s_{i-1}}\right]}^{k}\Delta s_{i-1}^{k}+{\left[\frac{\partial F_{i2}}{\partial p_{i-1}}\right]}^{k}\Delta p_{i-1}^{k}+{\left[\frac{\partial F_{i2}}{\partial s_{i}}\right]}^{k}\Delta s_{i}^{k}+{\left[\frac{\partial F_{i2}}{\partial p_{i}}\right]}^{k}\Delta p_{i}^{k}\;\;+{\left[\frac{\partial F_{i2}}{\partial s_{i+1}}\right]}^{k}\Delta s_{i+1}^{k}+{\left[\frac{\partial F_{i2}}{\partial p_{i+1}}\right]}^{k}\Delta p_{i+1}^{k}+F_{i2}^{k}=0,\;(i=2:N_{x}-1)$$

(41) $${\left[\frac{\partial F_{i1}}{\partial s_{i}}\right]}^{k}\Delta s_{i}^{k}+{\left[\frac{\partial F_{i1}}{\partial p_{i}}\right]}^{k}\Delta p_{i}^{k}+{\left[\frac{\partial F_{i1}}{\partial s_{i+1}}\right]}^{k}\Delta s_{i+1}^{k}+{\left[\frac{\partial F_{i1}}{\partial p_{i+1}}\right]}^{k}\Delta p_{i+1}^{k}+F_{i1}^{k}=0,{\left[\frac{\partial F_{i2}}{\partial s_{i}}\right]}^{k}\Delta s_{i}^{k}+{\left[\frac{\partial F_{i2}}{\partial p_{i}}\right]}^{k}\Delta p_{i}^{k}+{\left[\frac{\partial F_{i2}}{\partial s_{i+1}}\right]}^{k}\Delta s_{i+1}^{k}+{\left[\frac{\partial F_{i2}}{\partial p_{i+1}}\right]}^{k}\Delta p_{i+1}^{k}+F_{i2}^{k}=0,\;(i=1)$$

(42) $${\left[\frac{\partial F_{i1}}{\partial s_{i-1}}\right]}^{k}\Delta s_{i-1}^{k}+{\left[\frac{\partial F_{i1}}{\partial p_{i-1}}\right]}^{k}\Delta p_{i-1}^{k}+{\left[\frac{\partial F_{i1}}{\partial s_{i}}\right]}^{k}\Delta s_{i}^{k}+{\left[\frac{\partial F_{i1}}{\partial p_{i}}\right]}^{k}\Delta p_{i}^{k}+F_{i1}^{k}=0,{\left[\frac{\partial F_{i2}}{\partial s_{i-1}}\right]}^{k}\Delta s_{i-1}^{k}+{\left[\frac{\partial F_{i2}}{\partial p_{i-1}}\right]}^{k}\Delta p_{i-1}^{k}+{\left[\frac{\partial F_{i2}}{\partial s_{i}}\right]}^{k}\Delta s_{i}^{k}+{\left[\frac{\partial F_{i2}}{\partial p_{i}}\right]}^{k}\Delta p_{i}^{k}+F_{i2}^{k}=0,\;(i=N_{x})$$

In Eqs. (40)–(42), the notation *k* indicates that the function and partial derivatives of the functions are evaluated using the estimates of the solution at iteration *k* (at k=0, the solution at time step *n* is used). The above linear system of equations is then solved with respect to the changes in solution estimates defined by

(43) $$\Delta s_{i}^{k}=s_{i}^{k+1}-s_{i}^{k},\;\Delta p_{i}^{k}=p_{i}^{k+1}-p_{i}^{k},\;(i=1:N_{x})$$

After calculating the gradients in Eq. (43), they are used to update the solution estimates using Eq (44):

(44) $$s_{i}^{k+1}=\Delta s_{i}^{k}+s_{i}^{k},\;p_{i}^{k+1}=\Delta p_{i}^{k}+p_{i}^{k},\;(i=1:N_{x})$$

The coefficients and function values are updated, and the calculation is repeated iteratively until an acceptable error is reached. For simplicity of notation, we define the solution vector in the linear system as $\overset{\to }{y}=(y_{1},y_{2},\ldots ,y_{2N_{x}})$ and note that $y_{2i-1}=\Delta s_{i}^{k}$ and $y_{2i}=p_{i}^{k}$. There are hence $2N_{x}$ unknowns for a grid with $N_{x}$ cells and the linear system of equations gives rise to a hexagonal coefficient matrix where cell *i* has equations on rows 2i−1 and 2*i* of the matrix with coefficients for the six unknowns from $y_{2i-3}$ to $y_{2i+2}$. A summary of the algorithm for solving the above equations in pseudocode format is shown in Algorithm 1.

It should be noted that the specific structure of this system allows a direct solution approach if matrix operations are applied to the 2x2 coefficient matrices of each grid cell. In this work, the equations are applied in the Python programming language (Python 3.8.12). A basic version of the developed code is publicly available in the GitHub repository of the authors, under Apache License 2.0, by the link: https://github.com/jcabbasi/centrifuge_public.

## Appendix B Validation and quality check of the numerical model

In this appendix, the developed numerical models are validated. At first, it is tried to find optimum grid cell numbers with minimized numerical dispersion errors. Both OEO and TEO setups are tested for a single rotational speed of 40 rad/s with different cell numbers of 10, 20, 30, 40, and 50. The results are shown in Fig. A1. It is observed that the cell numbers of 40 can give the best results with pretty enough precision in both setups. So, all simulations in this study are performed with this spatial resolution.

Afterward, it is intended to validate the code against a third-party source. So, the TEO case is compared against an industrial reference simulator: Sendra v2018.5 core-scale simulator. This tool supports predictions just for the TEO setup, so only this model was validated. The simulations are carried out at the same rock and fluid properties (Table 1 and *Pc_WW*) and same rotational speeds (20, 30, 40, and 50 rad/s). Fig. A2(left) shows the equilibrium saturation profiles of the wetting phase at all the rotational speeds. The similarity in the distributions is a good indication of the validity of the model developed in this work. Also, the trend of cumulative produced water volume versus time (Fig. A2(right)) shows that the model successfully predicted the dynamic flow regime of phases before reaching equilibrium. After successful validation of the base code, the code corresponding to the OEO setup was developed by extension of the TEO code.

## Appendix C History match for relative permeability determination

One of the main applications of the OEO setup presented in this work, as was discussed in previous sections, is the calculation of counter-current relative permeability values. This calculation is performed using history matching of the curve of the produced volume (water) versus time in comparison to the numerical model results. In this method, it is tried to optimize the parameters of the relative permeability correlation (e.g., Corey). In this section, it is intended to verify the hypothesis that the inverse calculation of the relative permeability curve by history matching of the production profile results in a unique and correct solution and compares the results obtained for both TEO and OEO cases. Also, it is tried to understand the advantage of running both cases in parallel to find an average relative permeability curve. Below assumptions was presumed in this hypothesis:

• The governing capillary pressure is the same in both TEO and OEO setups.
• Except for the relative permeability curve, all the required parameters are known before, including the capillary pressure curve.
• The used relative permeability correlation (here, Corey equation) can practically describe the multiphase flow behavior in the rock.
• The produced volume is possible to be recorded with adequate accuracy.

In this section, the basic (previously introduced) simulation model used in this study (with the mixed-wet capillary pressure curve) is used for history matching purposes. The simulation results for this case were assumed as the observed (experimental) data for the history matching of relative permeability.

### C.1 Sensitivity analysis

Before trying the optimization of the production profile, here it is tried to conduct a sensitivity analysis on the relative impact of changing Corey parameters on the production profile of the centrifuge test. Here, the values of the four parameters are increased by 20%, separately. Fig. A3 shows the results of changes in the production profile of TEO and OEO setups for $k_{w}^{⁎}$ and $k_{nw}^{⁎}$. Fig. A4 shows these variations for changing of $n_{w}$ and $n_{nw}$ variables. The statistical summary of the differences is shown in Table 3. Furthermore, the related discussions are provided in the main text.

### C.2 Optimization

The Corey parameters, including wetting and nonwetting saturation indexes ($n_{w}$, $n_{nw}$) and endpoint relative permeabilities ($k_{w}^{⁎}$, $k_{nw}^{⁎}$) are chosen as the optimization variables. These variables are fitted to observed production data (see Fig. A5). using the Particle Swarm Optimization (PSO) algorithm. This nature-inspired algorithm is only one of the evolutionary optimization methods that try to minimize the objective function by iterative improvement of the solutions. The method works by considering a population (called swarms) of the possible solutions (particles). The population of swarms moves through the search space until the optimum solution is found. The advantage of the PSO algorithm is its tolerance in non-homogeneous conditions. This algorithm is originally introduced by Kennedy and Eberhart (1995), but a large number of variants are introduced after that. The position of a particle from $x_{k}^{i}$ will be evolved to $x_{k+1}^{i}$ as:

(45) $$x_{k+1}^{i}=x_{k}^{i}+v_{k+1}^{i}$$

Where in Eq. (45) the $v_{k+1}^{i}$ is the movement velocity:

(46) $$v_{k+1}^{i}=v_{k}^{i}+c_{1}r_{1}(p_{k}^{i}-x_{k}^{i})+c_{2}r_{2}(p_{k}^{g}-x_{k}^{i})$$

The subscript *k* indicates the increment of a time. $p_{k}^{i}$ is the optimum position of the swarm *i* at time *k* so far, while $p_{k}^{g}$ represents the global optimum position for all swarms at time *k*. In Eq. (46), $r_{1}$ and $r_{2}$ are also random values between 0 and 1. Also, $c_{1}$ and $c_{2}$ are the cognitive and social scaling parameters, respectively. More information about the theoretical aspects related to this algorithm is provided in the work of Kameyama (2009). In this work, a Python PSO library called PySwarms is utilized (Miranda, 2018). The objective function for the minimization task was mean square error (MSE) between the observation and simulation curves of the recovery factor. Here, MSE is defined as:

(47) $$\text{MSE}=\frac{1}{n}\sum_{i=1}^{n}{\left({\overset{ˆ}{y}}_{i}-y_{i}\right)}^{2}$$

Where *n* is the number of points, ${\overset{ˆ}{y}}_{i}$ is the observed RF, and $y_{i}$ is the predicted (simulation) RF. Table A1 shows the meta parameters of the applied PSO algorithm and Table A2 provides information about the lower and higher look-up range of the PSO algorithm for the optimization variables.

The optimization was carried out to the level of 800 separate runs for each of the TEO and OEO cases. The final results were analyzed for the cases with the lowest errors for 3 scenarios: TEO setup, OEO setup, and simultaneous optimization of both setups (their errors were averaged). By simultaneous optimization of both setups, we mean that the optimization loss function was based on the minimization of the RF curve of both cases. Fig. A6 shows the best fitted relative permeability curves for each case. It is clear that for all cases the fitted relative permeabilities are reasonable in comparison to the real curves. Fig. A7 and Fig. A8 show above optimization process repeated for cases with different viscosity ratios of M=31 ($\mu_{w}=0.07$ cp, $\mu_{nw}=2.17$ cp) and M=0.3 ($\mu_{w}=0.7$ cp, $\mu_{nw}=0.21$ cp), respectively.
